# From Lockdown to Knockdown: The Return of Violence and Sport in Post-COVID Nasal Fractures †

**DOI:** 10.3390/cmtr19030034

**Published:** 2026-08-07

**Authors:** Anna Aydin, Lawik Revend, Doha Revend, Thomas Beikler, Oliver Schuck, Manfred Giese, Florian Dudde

**Affiliations:** 1 Department of Oral and Maxillofacial Surgery, Army Hospital Hamburg, Lesserstraße 180, 22049 Hamburg, Germany; 2 Department of Plastic Surgery, Army Hospital Berlin, Scharnhorststraße 13, 10115 Berlin, Germany; 3 Department of Otolaryngology–Head and Neck Surgery, Army Hospital Berlin, Scharnhorststraße 13, 10115 Berlin, Germany; 4 Department of Periodontics, Preventive and Restorative Dentistry, University Medical Center Hamburg-Eppendorf, Martinistraße 52, 20251 Hamburg, Germany

**Keywords:** nasal bone fracture, maxillofacial trauma, COVID-19 pandemic, post-pandemic comparison

## Abstract

Background: The coronavirus disease 2019 (COVID-19) pandemic altered nasal fracture epidemiology, with fewer outdoor injuries and more domestic accidents. This study compared intra-pandemic and post-pandemic nasal fracture patterns and their prevention implications. Materials and Methods: This retrospective study included patients treated for nasal fractures during February 2020–January 2021 (intra-pandemic) and February 2023–January 2024 (post-pandemic). The post-pandemic cohort was identified using the same eligibility criteria, variable definitions, coding framework, and electronic-record retrieval procedure as the previously published intra-pandemic cohort. The predefined objective was a direct comparison of these two matched periods; no pre-pandemic control group was included. Results: A total of 290 patients were analyzed (184 post-pandemic and 106 intra-pandemic). In exploratory unadjusted comparisons, sports accidents occurred in 29/184 (15.8%) post-pandemic versus 7/106 (6.6%) intra-pandemic cases (*p* = 0.023), road traffic incidents in 31/184 (16.8%) versus 7/106 (6.6%) (*p* = 0.041), interpersonal violence in 60/184 (32.6%) versus 20/106 (18.9%) (*p* = 0.009), and alcohol-related events in 37/184 (20.1%) versus 7/106 (6.6%) (*p* = 0.009). Falls, virus- or flu-associated syncope, and accidents at home were more frequent intra-pandemic. Because multiple comparisons were performed, findings with *p*-values near 0.05 should be regarded as nominal and hypothesis-generating. The unadjusted mean hospital stay was shorter post-pandemic; however, the study period was not independently associated with length of stay after adjustment. The post-pandemic period showed a borderline, non-significant association with violence-related fractures (odds ratio 1.76, 95% confidence interval 0.99–3.12; *p* = 0.051). Conclusion: The observed changes are compatible with a return of social, recreational, and traffic-related trauma patterns, although the absence of a pre-pandemic control group and the exploratory nature of subgroup analyses limit causal interpretation. Differences in hospital stay appear to be more closely related to patient-level characteristics than to the study period itself.

## 1. Introduction

Nasal bone fractures are among the most common facial injuries, accounting for a substantial proportion of maxillofacial trauma cases worldwide. They often result from direct blunt force trauma and are frequently associated with interpersonal violence, sports accidents, and road traffic incidents. Epidemiological patterns of such injuries are influenced by socio-behavioral factors, environmental conditions, and seasonal variations [1]. The outbreak of the COVID-19 pandemic in early 2020 led to unprecedented public health interventions, including lockdowns, restrictions on public gatherings, suspension of sporting events, and closure of entertainment venues [2]. These measures profoundly altered patterns of injury presentation in emergency departments. Multiple studies have reported a reduction in maxillofacial trauma incidence during lockdown periods, accompanied by shifts in causative mechanisms, from high-energy outdoor injuries toward domestic accidents and falls within the home environment [3,4]. During the intra-pandemic phase, decreased social interaction and restricted mobility led to a marked reduction in injuries related to sports, interpersonal violence, and road traffic accidents. Simultaneously, the proportion of injuries caused by household accidents, falls, and virus- or flu-related syncope increased [5]. These shifts reflect both the immediate impact of social restrictions and the indirect consequences of altered physical activity patterns, mental health stressors, and healthcare-seeking behaviours during the pandemic. The eventual relaxation of restrictions and return to pre-pandemic routines in late 2022 and early 2023 reintroduced societal conditions conducive to traditional injury mechanisms. Sporting events resumed, nightlife and hospitality venues reopened, and physical contact in both recreational and occupational settings increased. While this societal reopening was expected to restore pre-pandemic injury profiles, limited evidence exists regarding the exact nature and magnitude of such changes, particularly in relation to nasal bone fractures, which serve as a sensitive marker for patterns of interpersonal violence and sports-related trauma [4,6,7]. Given the relative ease of diagnosis through clinical examination and imaging, nasal bone fractures provide an effective model for studying trauma epidemiology [2,8]. Furthermore, classification systems such as the Kim et al. nasal fracture type categorization allow for consistent morphological assessment across time periods [9]. Understanding the evolving epidemiology of these injuries can inform both preventive strategies and healthcare resource allocation, particularly in the context of fluctuating societal restrictions [1,10]. Most existing studies on nasal bone fractures during the COVID-19 pandemic have focused on the contrast between pre-pandemic and intra-pandemic conditions. The novelty of the present study lies in the direct comparison of an established intra-pandemic nasal-fracture cohort with a methodologically harmonized post-pandemic cohort, thereby examining what changed after restrictions were removed. The post-pandemic phase, with its gradual return to regular social and recreational activities, has received less attention. Yet, understanding how injury mechanisms evolved after the lifting of restrictions is crucial for a comprehensive assessment of long-term epidemiological changes. The present study therefore aimed to compare the epidemiological characteristics, injury mechanisms, and treatment modalities of nasal bone fractures between the intra-pandemic period (February 2020–January 2021) and the post-pandemic period (February 2023–January 2024) at the German Army Hospital Hamburg. By analyzing differences in demographics, fracture patterns, associated injuries, and hospital management, we provide a comprehensive assessment of post-pandemic shifts in nasal fracture epidemiology and their clinical implications. Beyond describing post-pandemic recovery, this paired-period design may also provide a framework for future comparative studies of trauma patterns during and after temporary restrictions on population mobility and social activity. A pre-pandemic control group was not included because the predefined study question focused on the direct comparison of the previously established intra-pandemic cohort with a matched post-pandemic observation period. This design permits assessment of changes after the lifting of restrictions but cannot determine whether the post-pandemic pattern represents a complete return to the pre-pandemic baseline or reflects broader secular trends.

## 2. Materials and Methods

### 2.1. Study Design and Setting

This retrospective comparative study was conducted at the Department of Oral and Maxillofacial Surgery, Army Hospital Hamburg, a metropolitan trauma center providing care to both military personnel and civilian emergency patients. The analysis included all patients diagnosed with nasal bone fractures during two distinct 12-month periods: the intra-pandemic phase (1 February 2020–31 January 2021) and the post-pandemic phase (1 February 2023–31 January 2024). These intervals were chosen to represent, respectively, a period under strict COVID-19-related public health restrictions and a subsequent period without major pandemic-related limitations. The intra-pandemic dataset had been published previously and was reused as a fixed reference cohort for direct comparison with the newly collected post-pandemic data [6]. Reuse of this predefined cohort avoided retrospective redefinition of the reference sample and allowed the post-pandemic cohort to be assembled using the same case-identification procedure, inclusion and exclusion criteria, electronic record source, variable definitions, and coding framework. The novelty of the design is therefore the harmonized intra-pandemic versus post-pandemic comparison rather than a reanalysis of the earlier cohort in isolation. A pre-pandemic control group was outside the predefined study question and was not available within the same harmonized data framework. Accordingly, the study cannot distinguish a true return to pre-pandemic baseline from unrelated temporal or secular changes. Military or civilian status was not included in the harmonized comparison dataset and therefore could not be reported reliably as separate subgroup counts or injury-type distributions.

### 2.2. Inclusion and Exclusion Criteria

Patients were eligible if they had a diagnosis of nasal bone fracture confirmed by clinical examination and/or radiological imaging (conventional radiography or computed tomography), presented within the defined study period, and had complete documentation of demographic data, injury mechanism, fracture classification, associated injuries, and treatment modality. Exclusion criteria were age under 18 years, pathological fractures (e.g., due to neoplastic disease), and incomplete or missing clinical records. These eligibility criteria were applied identically to the intra-pandemic and post-pandemic cohorts. Analyses were performed as complete-case analyses; no statistical imputation was used. Consequently, the 290 included patients had complete data for the variables required for the reported analyses.

### 2.3. Data Collection

Patient data were retrieved from the same electronic hospital record system for both periods using an identical retrospective extraction and coding procedure. The dataset included the following variables: demographics (age, sex), fracture characteristics (classification according to Kim et al.: Type I = linear fracture without depression; Type II = unilateral depression with or without septal fracture; Type III = bilateral depression with or without septal fracture; Type IV = comminuted fracture), associated injuries (nasal septum fracture, concomitant facial fracture, concomitant soft tissue injury, concomitant dental trauma), and injury mechanisms (fall, sports accident, epileptic accident, virus- or flu-associated syncope, road traffic accident [car, bicycle, e-scooter, motorcycle], interpersonal violence, domestic violence, accident at home, alcohol-related fracture) [9]. Alcohol involvement was coded only when explicitly documented in the clinical record on the basis of patient or witness history, treating-clinician assessment, police or emergency-service documentation, or a measured blood alcohol concentration where available. A record without explicit documentation was treated as no documented alcohol involvement; this definition may underestimate exposure and creates a risk of non-differential or differential misclassification. Temporal variables included month, weekday versus weekend, and time of accident (morning: 08:00–15:59; evening: 16:00–23:59; night: 00:00–07:59). Hospital management data comprised type of treatment (operative: open reduction under general anaesthesia or closed reduction under local anaesthesia; conservative management), length of hospital stay (days), interval from trauma to presentation (days), and interval from trauma to surgery (days). Variable definitions and category assignments were harmonized across both cohorts before analysis. For prevention planning, these variables covered patient characteristics, potentially modifiable injury contexts and exposures, injury severity, temporal clustering, and healthcare utilization. This framework permits identification of injury-prone settings and time windows, although exact geographic accident locations were not available for spatial hotspot analysis.

### 2.4. Statistical Analysis

Data analysis was performed using IBM SPSS Statistics, Version 28.0 (IBM Corp., Armonk, NY, USA). Continuous variables were assessed for normality using the Shapiro–Wilk test. Normally distributed variables are presented as mean ± standard deviation (SD) and were compared using the independent-samples *t*-test. Categorical variables are presented as absolute values and percentages and were compared using Pearson’s chi-square test or Fisher’s exact test, as appropriate. All tests were two-tailed. Because a large number of related categorical and subgroup comparisons were performed, these analyses were considered exploratory. Reported *p*-values are nominal and unadjusted for multiplicity; findings close to *p* = 0.05 were interpreted as suggestive and hypothesis-generating rather than confirmatory, with greater emphasis on effect size, consistency, and findings with very small *p*-values. The study used a census of all eligible cases in two predefined 12-month periods; therefore, no a priori sample-size calculation was performed. Precision is conveyed using 95% confidence intervals (CI), and the regression models were deliberately parsimonious to reduce overfitting. Exploratory multivariable analyses assessed potential predictors of (i) interpersonal violence as a cause of nasal fracture (binary outcome, logistic regression) and (ii) length of hospital stay (continuous outcome, linear regression). Pandemic period, sex, age, and documented alcohol involvement were selected a priori because they were clinically plausible, consistently available in both cohorts, and relevant to both outcomes. The restricted covariate set was chosen in view of the number of violence-related events and the overall sample size. Potential confounders not available or not included in the harmonized dataset included comorbidity burden, anticoagulant use, socioeconomic factors, detailed injury severity, referral source, changes in admission or discharge policy, and population exposure to specific transport modes. Results are presented as odds ratios (OR) with 95% CI for logistic regression and regression coefficients (β) with 95% CI for linear regression.

Model fit for the logistic regression was assessed using the omnibus likelihood-ratio test and the Hosmer–Lemeshow goodness-of-fit test. Explained variance was evaluated using Cox–Snell, Nagelkerke, and McFadden pseudo-R^2^, and discrimination was assessed using the area under the receiver operating characteristic curve. Multicollinearity was evaluated using variance inflation factors. Studentized residuals, leverage values, and Cook’s distances were examined to identify influential observations. For the linear regression, explained variance was reported using R^2^ and adjusted R^2^. Residual normality and homoscedasticity were evaluated using the Shapiro–Wilk, Breusch–Pagan, and White tests.

## 3. Results

**Figure 1:**
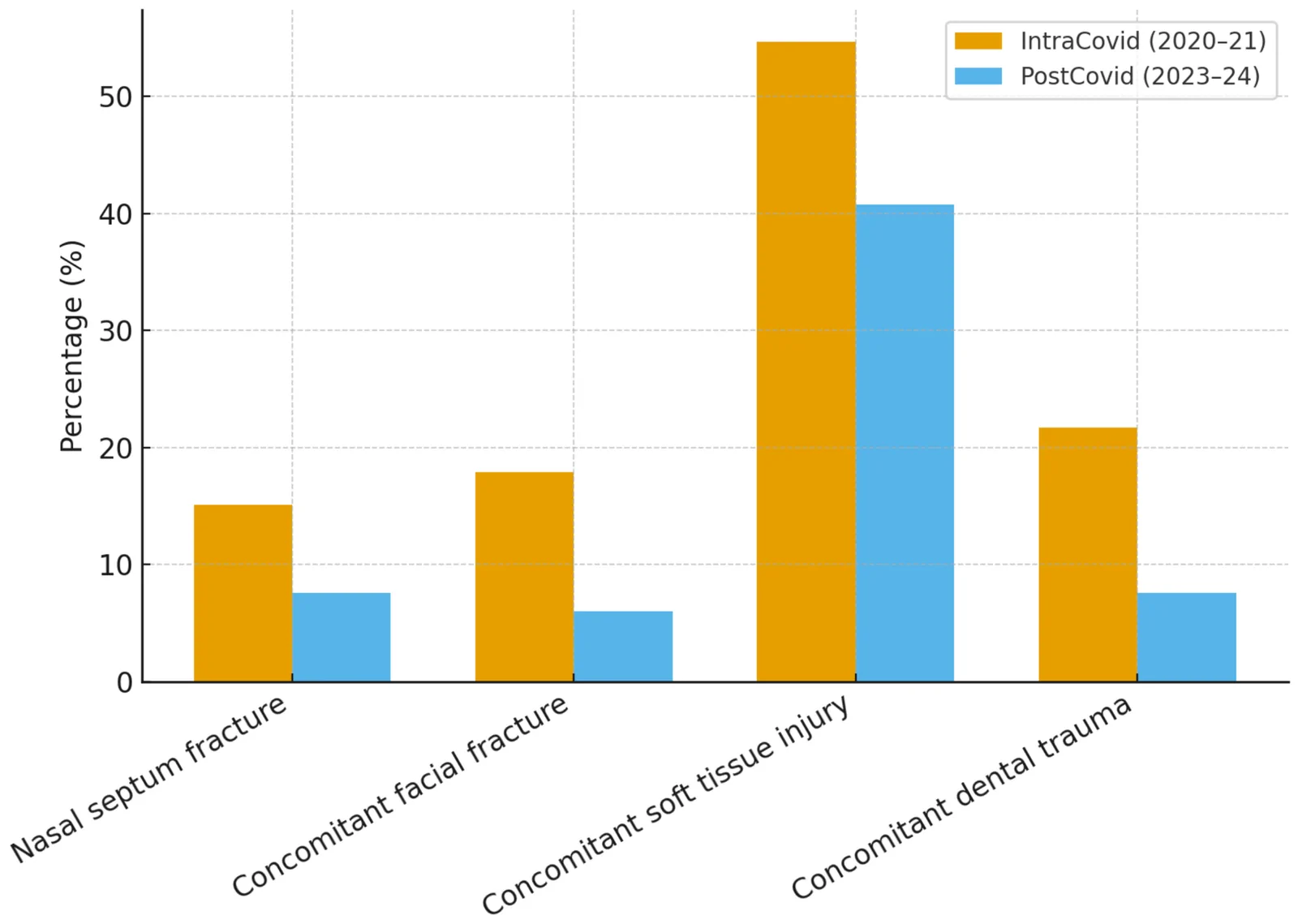
Associated injuries in nasal fractures (intra-COVID vs. post-COVID).

A total of 290 patients fulfilled the inclusion criteria, comprising 184 cases in the post-pandemic period and 106 cases in the intra-pandemic period. The mean age was 55.99 ± 25.85 years post-pandemic and 57.64 ± 25.50 years intra-pandemic (*p* = 0.600) (Table 1). Male patients accounted for 102/184 (55.4%) and 60/106 (56.6%), respectively (*p* = 0.847). Fracture morphology according to the Kim et al. classification did not show a clear overall difference (*p* = 0.227) [9] (Table 2). Type I fractures occurred in 97/184 (52.7%) post-pandemic and 69/106 (65.1%) intra-pandemic cases; Type II fractures occurred in 59/184 (32.1%) and 24/106 (22.6%), respectively. Unadjusted comparisons suggested higher intra-pandemic proportions of nasal septum fractures (16/106 [15.1%] vs. 14/184 [7.6%], *p* = 0.044) and soft tissue injuries (58/106 [54.7%] vs. 75/184 [40.8%], *p* = 0.022); these were nominal exploratory findings. More pronounced differences were observed for concomitant facial fractures (19/106 [17.9%] vs. 11/184 [6.0%], *p* = 0.001) and dental trauma (23/106 [21.7%] vs. 14/184 [7.6%], *p* < 0.001) (Table 2, Figure 1). The cohort included both military and civilian patients; however, personnel status was not part of the harmonized comparison dataset, so separate subgroup numbers and injury-type distributions could not be analyzed reliably.

**Figure 2:**
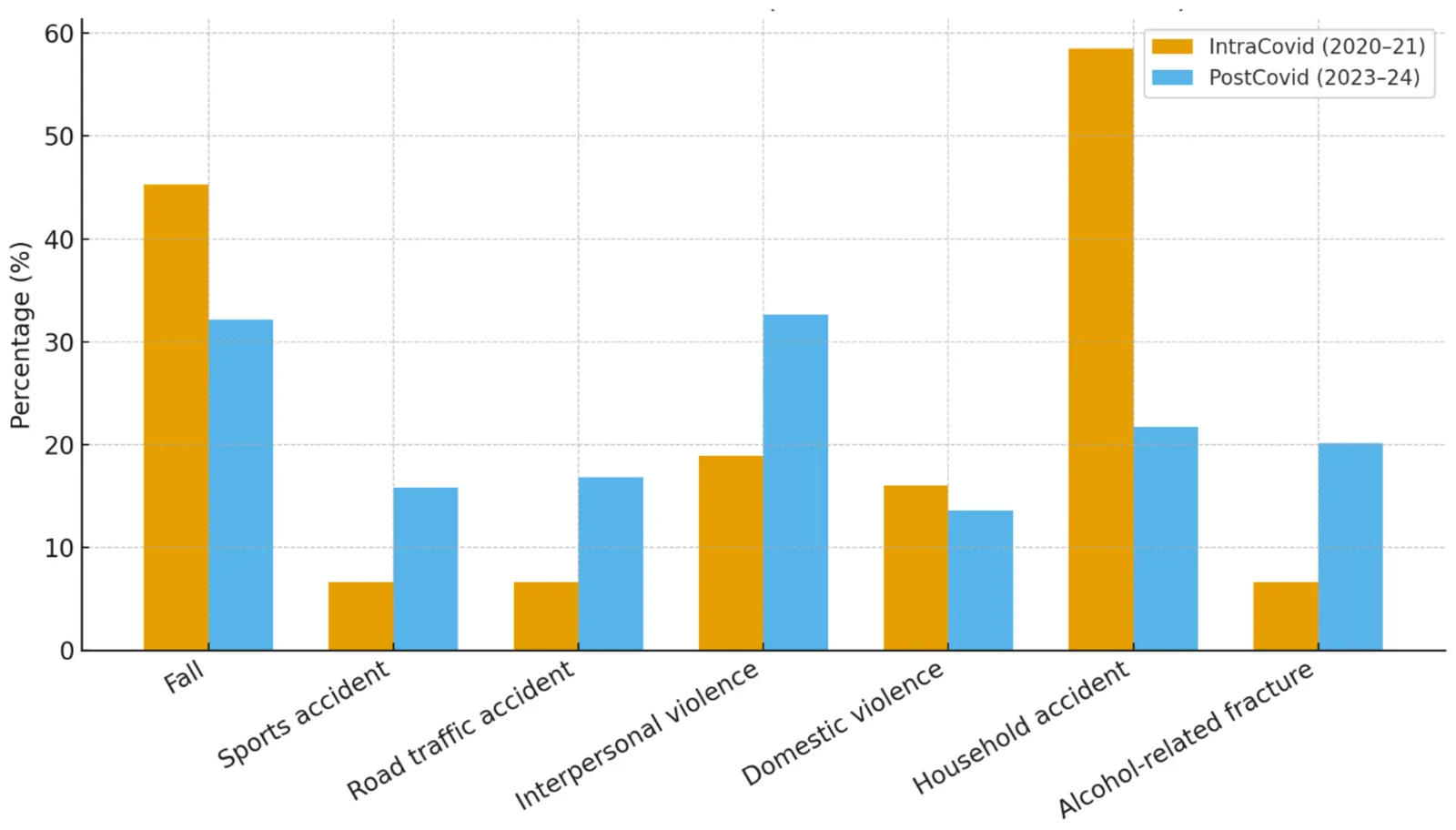
Causes of nasal bone fractures.

The distribution of injury mechanisms differed between the two observation periods. Falls occurred in 48/106 (45.3%) intra-pandemic and 59/184 (32.1%) post-pandemic cases (*p* = 0.039) (Table 3, Figure 2). Sports-related accidents occurred in 29/184 (15.8%) post-pandemic versus 7/106 (6.6%) intra-pandemic cases (*p* = 0.023), and road traffic accidents in 31/184 (16.8%) versus 7/106 (6.6%) (*p* = 0.041). These nominal differences are compatible with the resumption of organized sports, recreational activities, and mobility but require cautious interpretation. E-scooter-related injuries were recorded in 8/184 (4.3%) post-pandemic and 0/106 (0%) intra-pandemic cases (*p* = 0.042), likewise an exploratory subgroup finding. Interpersonal violence accounted for 60/184 (32.6%) and 20/106 (18.9%) cases (*p* = 0.009), whereas domestic violence occurred in 25/184 (13.6%) and 17/106 (16.0%) (*p* = 0.341). Alcohol-related fractures were documented in 37/184 (20.1%) post-pandemic and 7/106 (6.6%) intra-pandemic cases (*p* = 0.009). The most robust differences concerned virus- or flu-associated syncope (20/106 [18.8%] intra-pandemic vs. 5/184 [2.7%] post-pandemic, *p* < 0.001) and accidents at home (62/106 [58.5%] vs. 40/184 [21.7%], *p* < 0.001) (Table 3).

Temporal analysis showed a clear difference in the distribution of accident times. Morning injuries occurred in 66/106 (62.3%) intra-pandemic versus 40/184 (21.7%) post-pandemic cases, whereas evening injuries occurred in 31/106 (29.2%) and 114/184 (61.9%), respectively (both *p* < 0.001) (Table 3). Night-time injuries were observed in 30/184 (16.3%) post-pandemic and 9/106 (8.5%) intra-pandemic cases (*p* = 0.047), and weekend injuries in 82/184 (44.6%) and 33/106 (31.1%), respectively (*p* = 0.043). The latter findings are nominal and should be interpreted cautiously in view of multiple testing. No difference was observed in the overall monthly distribution of cases (*p* = 0.988) (Table 3). With regard to hospital management, the mean interval from trauma to presentation was 0.34 ± 1.29 days post-pandemic and 0.58 ± 1.02 days intra-pandemic (*p* = 0.154) (Table 4). The time from trauma to surgical intervention was 0.37 ± 1.30 versus 0.38 ± 1.28 days (*p* = 0.878). The previously reported unadjusted comparison showed a shorter mean hospital stay post-pandemic (0.58 ± 2.55 vs. 1.35 ± 2.16 days, *p* < 0.001) (Table 4). Because hospital stay showed a right-skewed distribution, a Mann–Whitney U test was additionally performed and confirmed this finding (U = 2346.5, Z = −5.43, *p* < 0.001) (Table 4).

To account for potential confounding, exploratory multivariable regression analyses were performed. In the logistic model for interpersonal violence, the post-pandemic period showed a borderline association that did not reach the conventional threshold for statistical significance (OR 1.76, 95% CI 0.99–3.12; *p* = 0.051); age, sex, and documented alcohol involvement were also not independently significant (Table 5). In the linear model for length of hospital stay, older age (β = +0.012 days/year, 95% CI 0.001–0.023; *p* = 0.036) and alcohol involvement (β = +0.82 days, 95% CI 0.07–1.58; *p* = 0.033) were associated with longer admission, whereas study period was not independently associated with length of stay (β = −0.42 days, 95% CI −0.99 to 0.16; *p* = 0.155) (Table 5). The logistic regression showed acceptable calibration (Hosmer–Lemeshow χ^2^(8) = 7.77, *p* = 0.456), and the omnibus likelihood-ratio test was significant (χ^2^(4) = 12.79, *p* = 0.012). Variance inflation factors ranged from 1.01 to 1.04, indicating no relevant multicollinearity. Model explanatory power was limited (Cox–Snell R^2^ = 0.043, Nagelkerke R^2^ = 0.062, and McFadden R^2^ = 0.037), and discrimination was modest (ROC AUC = 0.639). No extreme studentized residuals were identified, and the maximum Cook’s distance was 0.030.

The linear model explained 8.6% of the variance in length of hospital stay (adjusted R^2^ = 0.073; F(4285) = 6.70, *p* < 0.001). Although multicollinearity was negligible, residual analysis demonstrated marked non-normality, heteroskedasticity, and at least one highly influential observation. The linear-model results were therefore interpreted cautiously.

## 4. Discussion

This study provides a comparative analysis of nasal bone fracture epidemiology during the intra-pandemic and post-pandemic periods, identifying differences in injury mechanisms, associated trauma patterns, and temporal distribution. Because numerous related outcomes and subgroups were examined, the unadjusted comparisons are interpreted as exploratory. The findings nevertheless suggest that the lifting of public health restrictions coincided with a return of social, recreational, and mobility-related injury patterns. The use of matched 12-month observation windows and a harmonized variable framework also provides a reference model for future comparative studies examining trauma epidemiology during and after temporary restrictions on population activity.

The similar age and sex distributions between study periods make major demographic shifts an unlikely sole explanation for the observed patterns, although residual confounding cannot be excluded. The overall male predominance is consistent with prior studies on maxillofacial trauma, which attribute this disparity to greater male participation in high-risk activities, contact sports, and interpersonal altercations [11,12]. Fracture morphology according to the Kim classification remained broadly stable [9]. Unadjusted comparisons suggested lower post-pandemic proportions of concomitant facial fractures, soft tissue injuries, dental trauma, and nasal septum fractures. The apparently greater associated-injury burden intra-pandemic, despite fewer conventionally high-energy outdoor mechanisms, may reflect the characteristics of falls and syncope in an older population, impacts against hard indoor surfaces, delayed presentation, or selective referral of more complex cases during periods of restricted healthcare access. It may also reflect differences in documentation or unmeasured injury severity. Mechanism categories therefore should not be interpreted as direct proxies for impact energy, and the retrospective data cannot establish which explanation predominated.

The most striking differences were observed in the mechanisms of injury. The intra-pandemic period was characterised by a predominance of low-energy, domestic mechanisms specifically, falls and virus/flu-associated syncope. This pattern aligns with previously reported reductions in outdoor activities, closure of sports facilities, and increased time spent indoors during lockdown phases. Comparable findings have been reported in studies from the United Kingdom, Italy, and South Korea, where domestic accidents and falls replaced sports injuries and road traffic incidents as leading causes of nasal fractures during lockdown periods [4,6,13,14].

In the post-pandemic period, higher proportions of outdoor and social injury mechanisms were observed. Sports accidents more than doubled in the unadjusted comparison, consistent with the resumption of organised sports leagues, recreational training, and fitness activities [15]. Road traffic accidents were also more frequent, particularly those involving bicycles and e-scooters [15,16]. However, the *p*-values for sports accidents, road traffic accidents, and the e-scooter subgroup were close to 0.05 and should therefore be interpreted as nominal exploratory evidence rather than definitive proof of period effects. The occurrence of e-scooter-related fractures only in the post-pandemic cohort is nevertheless compatible with reports from other urban European settings describing increasing e-scooter trauma, often in association with evening use and alcohol consumption [17,18].

Interpersonal violence increased from 20/106 (18.9%) during the pandemic to 60/184 (32.6%) post-pandemic in the unadjusted analysis. Several maxillofacial trauma studies have described similar post-reopening patterns following the return of bars, clubs, and large public gatherings [4,19,20,21,22]. Documented alcohol-related nasal fractures also increased from 7/106 (6.6%) to 37/184 (20.1%), indicating overlap between alcohol exposure and post-pandemic trauma circumstances, although the retrospective definition of alcohol involvement is vulnerable to under-documentation and misclassification and does not permit causal inference [23,24]. In the multivariable logistic model, the association between study period and violence-related fractures was borderline and did not reach conventional statistical significance (OR 1.76, 95% CI 0.99–3.12; *p* = 0.051). Accordingly, the data suggest, but do not independently confirm, that broader societal reopening contributed to the observed increase. The clear shift from morning injuries intra-pandemic to evening injuries post-pandemic is consistent with altered social and leisure activity. By contrast, the increases in night-time and weekend injuries had *p*-values close to 0.05 and should be viewed as supportive exploratory observations rather than conclusive evidence.

The unadjusted analysis showed a shorter mean hospital stay in the post-pandemic cohort. Although changes in outpatient pathways or discharge practice could theoretically contribute to this finding, the present data do not support interpreting study period as an independent operational driver. In the multivariable linear regression, the association with study period was attenuated and no longer statistically significant, whereas older age and alcohol involvement were associated with longer admission. The crude between-period difference therefore appears to be confounded, at least partly, by patient-level characteristics. Any inference regarding systemic hospital capacity, treatment efficiency, or discharge policy would require additional variables and a study design specifically addressing these factors.

From a public health perspective, the recorded variables allow prevention priorities to be linked to identifiable risk contexts. Sports mechanism, fracture morphology, and associated injury burden may guide protective equipment recommendations and enforcement of fair-play regulations. Bicycle and e-scooter mechanisms, alcohol involvement, and evening, night-time, or weekend clustering may inform urban mobility measures, venue-based prevention, and education on safe riding and responsible alcohol consumption. Falls, syncope, and accidents at home may support domestic fall-prevention measures and assessment of underlying medical risk factors. Addressing interpersonal violence requires a multifaceted approach involving community policing, venue safety measures, and prevention programs focused on high-risk time periods and settings [23,24]. Future multicenter studies should prospectively add military or civilian status, exact accident location, protective equipment use, and population exposure denominators to identify regional hotspots and tailor prevention strategies to specific populations and injury-prone areas.

Our results must be interpreted within the context of several limitations. The retrospective design is inherently dependent on the accuracy and completeness of medical records. Alcohol involvement may have been under-recorded or inconsistently documented, and no imputation was performed. The study was conducted at a major military hospital within a metropolitan region that also serves a substantial civilian catchment area; therefore, the findings may not be fully representative of the general population. Military or civilian status was not included in the harmonized dataset, which prevented reliable subgroup analysis. A pre-pandemic control group was not included, so the study cannot determine whether post-pandemic patterns represent a complete return to baseline rather than secular change. Changes in referral pathways, admission or discharge policies, healthcare-seeking behaviour, and hospital capacity could have differed between periods and were not measured. The expansion of e-scooter availability and use during the intervening years is a particularly important external exposure and may partly explain the observed post-pandemic e-scooter injuries independently of pandemic restrictions. Other omitted confounders include comorbidity, anticoagulation, socioeconomic factors, detailed injury severity, and exposure denominators. Exact geographic accident locations were unavailable, so region-specific injury hotspots and incidence rates could not be determined. The intra-pandemic period reflects the first major lockdown phase, and later phases with partial restrictions may have differed [25,26,27]. In addition, a large number of categorical outcomes and subgroup comparisons were examined without formal adjustment for multiple testing. The reported *p*-values are therefore nominal, and findings close to 0.05 may represent false-positive associations. The regression models were exploratory and parsimonious, and should therefore be interpreted cautiously. The logistic regression showed acceptable calibration but low explained variance and limited discrimination and should therefore be interpreted as an exploratory association model rather than as a clinical prediction model. The linear length-of-stay model showed substantial violations of conventional OLS assumptions, including non-normality, heteroskedasticity, and influential observations. Its findings should therefore be regarded as sensitivity analyses and interpreted cautiously. Future confirmatory studies should include a pre-pandemic baseline, prespecify primary outcomes, use multiplicity-adjusted approaches, and prospectively record referral source, geographic location, personnel status, protective equipment, alcohol testing, injury-severity measures, and relevant population exposures.

## 5. Conclusions

The transition from the intra-pandemic to the post-pandemic period was accompanied by changes in the observed distribution of nasal bone fracture mechanisms. Robust unadjusted differences were seen for virus- or flu-associated syncope and accidents at home, while higher post-pandemic proportions of sports-, traffic-, violence-, and documented alcohol-related trauma were exploratory observations. The adjusted association between the post-pandemic period and violence-related fractures was borderline and non-significant. Likewise, the shorter unadjusted hospital stay was not independently associated with study period after adjustment. These data are compatible with an influence of changing societal behaviour on trauma patterns but do not establish causality or a return to pre-pandemic baseline. Interpretation is limited by the absence of a pre-pandemic control group, multiple testing, potential exposure misclassification, and unmeasured changes in referral patterns, hospital policy, and transport exposure. Confirmatory multicenter studies with prespecified outcomes and broader covariate collection are required before definitive prevention or operational conclusions can be drawn.

## Figures and Tables

**Table 1:** Baseline characteristics.

Variable	Post-COVID (n = 184)	Intra-COVID (n = 106)	*p*-Value
Age (years)	55.99 ± 25.85	57.64 ± 25.50	0.600
Gender			0.847
Male	102 (55.4)	60 (56.6)	
Female	82 (44.6)	46 (43.4)	

Note: Continuous data are presented as mean ± SD; categorical data are presented as n (%).

**Table 2:** Nasal fracture patterns.

Variable	Post-COVID (n = 184)	Intra-COVID (n = 106)	*p*-Value
Nasal fracture type			0.227
I	97 (52.7)	69 (65.1)	
II	59 (32.1)	24 (22.6)	
III	23 (12.5)	11 (10.4)	
IV	5 (2.7)	2 (1.9)	
Nasal septum fracture	14 (7.6)	16 (15.1)	0.044
Concomitant facial fracture	11 (6.0)	19 (17.9)	0.001
Concomitant soft tissue injury	75 (40.8)	58 (54.7)	0.022
Concomitant dental trauma	14 (7.6)	23 (21.7)	<0.001
Operation	45 (24.5)	39 (36.8)	0.089
Open reduction under general anaesthesia	6 (3.3)	8 (7.5)	0.101
Closed reduction under local anaesthesia	39 (21.2)	31 (29.2)	0.123
Conservative treatment	139 (75.5)	67 (63.2)	0.089

Note: Data are presented as n (%). Nasal fracture type: Type I = linear fracture without depression; Type II = unilateral depression with or without septal fracture; Type III = bilateral depression with or without septal fracture; Type IV = comminuted fracture [9].

**Table 3:** Causes of nasal fracture.

Variable	Post-COVID (n = 184)	Intra-COVID (n = 106)	*p*-Value
Fall	59 (32.1)	48 (45.3)	0.039
Sports accident	29 (15.8)	7 (6.6)	0.023
Epileptic accident	0 (0)	4 (3.8)	0.254
Virus-/flu-associated syncope	5 (2.7)	20 (18.8)	<0.001
Road traffic accident	31 (16.8)	7 (6.6)	0.041
Car	4 (2.2)	2 (1.9)	0.869
Bicycle	19 (10.3)	4 (3.8)	0.123
E-Scooter	8 (4.3)	0 (0)	0.042
Motorcycle	0 (0)	1 (0.9)	0.783
Interpersonal violence	60 (32.6)	20 (18.9)	0.009
Domestic violence	25 (13.6)	17 (16.0)	0.341
Accident at home	40 (21.7)	62 (58.5)	<0.001
Alcohol-related fracture	37 (20.1)	7 (6.6)	0.009
Month			0.988
January	14 (7.6)	11 (10.4)	
February	11 (6.0)	10 (9.4)	
March	15 (8.2)	7 (6.6)	
April	18 (9.8)	8 (7.5)	
May	12 (6.5)	8 (7.5)	
June	18 (9.8)	9 (8.5)	
July	14 (7.6)	8 (7.5)	
August	16 (8.7)	10 (9.4)	
September	21 (11.4)	9 (8.5)	
October	15 (8.2)	9 (8.5)	
November	15 (8.2)	9 (8.5)	
December	15 (8.2)	8 (7.5)	
Weekday	102 (55.4)	73 (68.9)	0.043
Weekend	82 (44.6)	33 (31.1)	0.043
Daytime of accident			
Morning	40 (21.7)	66 (62.3)	<0.001
Evening	114 (61.9)	31 (29.2)	<0.001
Night	30 (16.3)	9 (8.5)	0.047
Work-related accident	10 (5.4)	7 (6.6)	0.878

Note: Data are presented as n (%). Morning = 08:00–15:59; evening = 16:00–23:59; night = 00:00–07:59.

**Table 4:** Hospital management variables.

Variable	Post-COVID (n = 184)	Intra-COVID (n = 106)	*p*-Value
Days from trauma to presentation	0.34 ± 1.29	0.58 ± 1.02	0.154
Days from trauma to surgery	0.37 ± 1.30	0.38 ± 1.28	0.878
Length of hospital stay (days)	0.58 ± 2.55	1.35 ± 2.16	<0.001

Note: Data are presented as mean ± SD. Because length of hospital stay showed a right-skewed distribution, the between-group comparison was additionally confirmed using a Mann–Whitney U test (U = 2346.5, Z = −5.43, *p* < 0.001).

**Table 5:** Multivariate regression analyses of predictors for interpersonal violence and hospital stay.

Predictor	OR (95% CI)	*p*	β (95% CI)	*p*
Post- vs. intra-COVID	1.76 (0.99–3.12)	0.051	−0.42 (−0.99 to 0.16)	0.155
Male sex	0.85 (0.50–1.44)	0.547	−0.39 (−0.95 to 0.17)	0.166
Age (per year)	0.99 (0.98–1.00)	0.134	+0.012 (0.001 to 0.023)	0.036
Alcohol involvement	0.83 (0.40–1.71)	0.607	+0.82 (0.07 to 1.58)	0.033

Note: OR = odds ratio; β = regression coefficient; CI = confidence interval.

## Data Availability

The data presented in this study are not publicly available due to ethical, legal, and institutional restrictions related to patient confidentiality and data protection. The data originate from a German Bundeswehr hospital and are subject to strict institutional governance and German data protection regulations. Therefore, the datasets generated and/or analyzed during the current study are not available for public sharing. A generative artificial intelligence-assisted language model was used to support English-language editing and restructuring. It was not used to collect data, perform statistical analyses, or make autonomous scientific decisions.
